# The growing importance of radiation worker studies

**DOI:** 10.1038/s41416-018-0134-6

**Published:** 2018-08-15

**Authors:** Richard Wakeford

**Affiliations:** Centre for Occupational and Environmental Health, The University of Manchester, Manchester, UK

**Keywords:** Cancer epidemiology, Risk factors

## Abstract

Large radiation worker studies have the potential to provide precise risk estimates for protracted exposure to low-level ionising radiation. Recent worker studies have reported statistically discernible dose-related increased risks of cancer; however, results must be interpreted with care, and occupational radiation doses need to be treated with particular attention.

## Main

The dose limits and constraints used to protect workers and members of the general public against the health effects that are potentially caused by low-level exposure to ionising radiation, are based primarily upon the findings of the impressive epidemiological studies of the Japanese survivors of the atomic bombings of Hiroshima and Nagasaki in 1945.^1,2^ Much of the evidence on radiation-related diseases in these atomic-bomb survivors, who were briefly exposed to mainly gamma-rays, stems from those who received moderate or high doses. This begs the question of how this information may be used appropriately to derive risk estimates for low doses, and doses received at low dose-rates.

The conventional approach for the purposes of radiological protection, at least for cancer and hereditary disease, is the use of the linear no-threshold (LNT) dose-response model. This model posits that the excess risk is directly proportional to the received dose, with no threshold dose below which there is zero risk. The LNT model is assumed to be prudent and conservative, but not overly so.^1,2^ However, it is essential to test this assumption by investigating other exposure circumstances. Studies of radiation workers provide one important approach, because these workers are generally exposed to a series of low doses received at low dose-rates over protracted periods (often years). Nonetheless, because most workers receive low cumulative doses, studies must include large numbers to achieve sufficient power to produce meaningful results. The latest radiation worker study is that of Haylock et al.,^3^ updating the follow-up of those included in the National Registry for Radiation Workers (NRRW), which has been collecting and collating data on radiation workers in the UK since 1976.

The study of Haylock et al.^3^ represents an important advance in providing evidence of increased cancer risks consequent to protracted low-level exposure to radiation. The study is notable because of the large numbers (nearly 170,000) included in the study, and because the workers are largely White British men who experience background risks for many cancer sites that are quite different from those of the atomic-bomb survivors exposed in mid-twentieth century Japan, addressing the key issue of how radiation-related risks differ between populations. This latest NRRW study may have only extended the follow-up by 10 years when compared with the previous report,^4^ but the additional cancer deaths and incident cases now included in the analysis are predominantly among earlier workers who tended to receive higher lifetime doses. It is these workers who carry much of the information on the effects of protracted occupational exposure to radiation. Only ~6% (~10,500) of workers included in the NRRW received individual cumulative doses ≥100 mSv (the conventional boundary between low and moderate doses); however, they provide ~60% of the collective dose, which under the LNT model represents the collective excess risk. This exemplifies why it is important to continue the follow-up of the NRRW workers: of those who received cumulative doses ≥100 mSv, 43% died before the end of this latest follow-up. This is a significant increase on the previous NRRW study and provides greater statistical power, which is reflected in the narrower confidence intervals for the raised solid cancer risk estimates presented by Haylock et al.^3^ Though, most of the evidence on cancer in workers in the NRRW with cumulative doses ≥100 mSv is yet to be revealed with future studies.

The NRRW is just one of a number of databases around the world that includes workers involved in early operations at nuclear establishments; particularly those workers employed when there was pressure to quickly produce materials for nuclear weapons, a period when many workers were exposed to radiation levels that would not be acceptable today. An extreme manifestation of this was the Mayak plutonium production plant in the former USSR, where workers were exposed to high levels of external radiation and plutonium.^5^ The pooling of such datasets extracts the maximum information from workers who received moderate and high cumulative doses at low dose-rates, and a recent example is the pooled analysis of plutonium workers from the Mayak and Sellafield nuclear installations.^6,7^ The NRRW is an important component of the latest international radiation worker collaboration, the International Nuclear Workers Study (INWORKS), which also includes French nuclear workers and those from six installations in the USA. Both the latest NRRW study and INWORKS provide evidence that occupational exposure to radiation increases the subsequent risk of cancer, but to an extent that is comparable with that predicted from the risk estimates obtained from the Japanese atomic-bomb survivors. These findings thus provide reassurance that the present framework of radiological protection is on a reasonably secure footing. These studies also provide evidence that the LNT model is an acceptable approximation of excess cancer risk following protracted exposure to low dose-rates.

One of the advantages of nuclear worker studies is that, in contrast to the atomic-bomb survivors, most workers were monitored for exposure to external sources of radiation, such that individual dose records are available for epidemiological studies. However, doses were measured for the purposes of radiological protection and the direct use of these records is not necessarily appropriate for epidemiology. By way of illustration, “missed” photon doses occurred when early dosimeters had fairly high limits of detection (LoD), were changed frequently, and doses below the LoD were recorded as zero; thus, non-trivial doses could be hidden by the monitoring and recording practices at the time. In the series of NRRW studies, certain adjustments have been made to the recorded doses to provide better estimates of the doses actually received by the workers; however, these adjustments are especially difficult for doses recorded in the crucial early years of the nuclear industry.

**Fig. 1:**
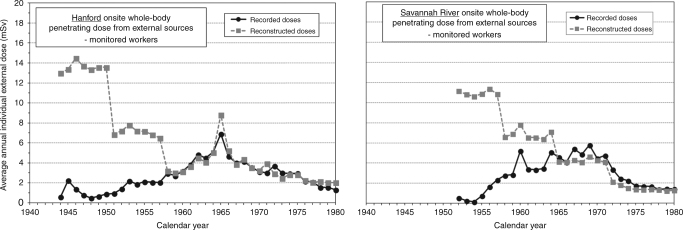
Comparison of the distributions by calendar year of average annual individual whole-body doses received occupationally from penetrating (primarily photon) radiation from external sources, as recorded at the US nuclear installations at Hanford^13^ and Savannah River,^14^ and as reconstructed^8^ for the purposes of compensating people who develop cancers that could be attributable to prior occupational exposure to radiation at these installations

There are other dosimetry issues to be considered, such as doses from unmonitored exposures to neutrons and from intakes of radioactive materials such as plutonium. Figure 1 indicates the potential for the underestimation of external penetrating radiation doses, in the early years of operations at two US nuclear installations with workers included in INWORKS. Undoubtedly, the claimant-favourable assumptions made by Merwin et al.,^8^ when reconstructing doses for the purposes of worker compensation, led to unrecorded doses being overestimated; possibly substantially so. Nonetheless, the differences between recorded and reconstructed doses shown in Fig. 1 illustrate the importance of properly accounting for dosimetry uncertainties, particularly in the early years (and it will be noted that recorded and reconstructed doses are similar in later years). Failure to appropriately adjust occupational doses for measurement and recording deficiencies may lead to biased risk estimates, as investigated in the UK by Inskip et al.^9^ and in the USA by Frome et al.,^10^ the latter study suggesting that failure to adjust for missed photon doses would lead to inflated risk estimates. INWORKS currently only includes doses received from external sources of photons, with some dose adjustments to account for certain factors (such as shielding of deep tissues by the body); doses received from neutrons and internally deposited radionuclides were excluded. It is feasible that these excluded doses and unrecorded doses may be positively correlated with the photon doses used in the INWORKS analyses. Standards of occupational hygiene in the early years of the nuclear industry may not only have led to higher recorded photon doses during this period, but also to higher neutron doses and intakes of radioactive materials, as well as providing the greatest potential for missed doses. Such a positive correlation would lead to an overestimation of the slope (risk per unit dose) of the dose-response for the photon doses presently used in INWORKS, and this must be borne in mind when assessing the INWORKS results.

There is no doubt that epidemiological studies of radiation workers will increase our understanding of the health effects of protracted exposure to low-level radiation, and the continuing follow-up of early workers will particularly increase the precision of risk estimates. Efforts in the USA to study large numbers of workers exposed to low levels of radiation could add substantially to the available evidence,^11^ especially when contributing to international collaborations. Nonetheless, as ever, caution is required in the interpretation of epidemiological studies, and the puzzling patterns of dose-related cancer risks found in the study of an important subset of workers within the NRRW are a reminder of this.^12^ Radiation worker studies have great potential, but insight into the complexities hidden within the data is essential if appropriate adjustments are to be made and findings are to be properly interpreted.
